# 5-(Adamantan-1-yl)-3-(benzyl­sulfan­yl)-4-methyl-4*H*-1,2,4-triazole

**DOI:** 10.1107/S1600536812030784

**Published:** 2012-07-10

**Authors:** Ebtehal S. Al-Abdullah, Ali A. El-Emam, Hazem A. Ghabbour, Suchada Chantrapromma, Hoong-Kun Fun

**Affiliations:** a Department of Pharmaceutical Chemistry, College of Pharmacy, King Saud University, PO Box 2457, Riyadh 11451, Saudi Arabia; b Crystal Materials Research Unit, Department of Chemistry, Faculty of Science, Prince of Songkla University, Hat-Yai, Songkhla 90112, Thailand; c X-ray Crystallography Unit, School of Physics, Universiti Sains Malaysia, 11800 USM, Penang, Malaysia

## Abstract

In the asymmetric unit of the title adamantyl derivative, C_20_H_25_N_3_S, there are two crystallographic independent mol­ecules with slightly different conformations. In one mol­ecule, the whole benzyl group is disordered over two orientations with the refined site-occupancy ratio of 0.63 (2):0.37 (2). The dihedral angles between the 1,2,4-triazole and phenyl rings are 24.3 (8) (major component) and 25.8 (13)° (minor component) in the disordered mol­ecule, whereas the corresponding angle is 51.53 (16)° in the other mol­ecule. In the crystal, mol­ecules are linked into a chain along the *a* axis by a weak C—H⋯N inter­action. Weak C—H⋯π inter­actions are also observed.

## Related literature

For bond-length data, see: Allen *et al.* (1987 ▶). For the synthesis and biological activity of adamantyl-1,2-4-triazole derivatives, see: El-Emam & Ibrahim (1991 ▶); El-Emam *et al.* (2004 ▶); Kadi *et al.* (2007 ▶, 2010 ▶); Togo *et al.* (1968 ▶). For related adamantyl-1,2,4-triazole structures, see: Al-Abdullah *et al.* (2012 ▶); El-Emam *et al.* (2012 ▶). For a substituted sulfanyl-1,2,4-triazole structure, see: Fun *et al.* (2011 ▶).
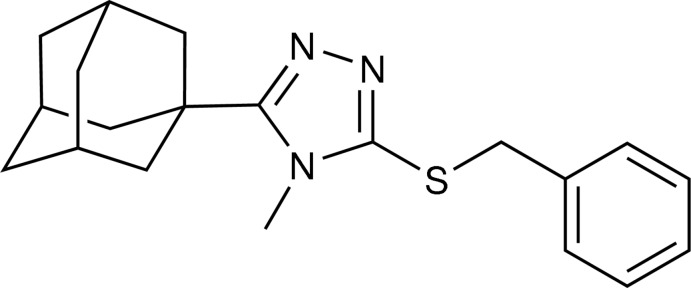


## Experimental

### 

#### Crystal data

C_20_H_25_N_3_S
*M* *_r_* = 339.49
Triclinic, 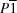

*a* = 6.4554 (3) Å
*b* = 14.0258 (6) Å
*c* = 20.2264 (9) Å
α = 94.610 (2)°
β = 95.568 (3)°
γ = 98.317 (3)°
*V* = 1795.23 (14) Å^3^
*Z* = 4
Cu *K*α radiation
μ = 1.63 mm^−1^
*T* = 296 K
0.94 × 0.12 × 0.07 mm

#### Data collection

Bruker SMART APEXII CCD area-detector diffractometer
Absorption correction: multi-scan (*SADABS*; Bruker, 2009 ▶) *T*_min_ = 0.310, *T*_max_ = 0.900
22288 measured reflections
6505 independent reflections
4522 reflections with *I* > 2σ(*I*)
*R*_int_ = 0.081

#### Refinement

*R*[*F* ^2^ > 2σ(*F* ^2^)] = 0.052
*wR*(*F* ^2^) = 0.143
*S* = 1.03
6505 reflections
500 parameters
15 restraints
H-atom parameters constrained
Δρ_max_ = 0.30 e Å^−3^
Δρ_min_ = −0.26 e Å^−3^

### 

Data collection: *APEX2* (Bruker, 2009 ▶); cell refinement: *SAINT* (Bruker, 2009 ▶); data reduction: *SAINT*; program(s) used to solve structure: *SHELXTL* (Sheldrick, 2008 ▶); program(s) used to refine structure: *SHELXTL*; molecular graphics: *SHELXTL*; software used to prepare material for publication: *SHELXTL* and *PLATON* (Spek, 2009 ▶).

## Supplementary Material

Crystal structure: contains datablock(s) global, I. DOI: 10.1107/S1600536812030784/is5162sup1.cif

Structure factors: contains datablock(s) I. DOI: 10.1107/S1600536812030784/is5162Isup2.hkl

Supplementary material file. DOI: 10.1107/S1600536812030784/is5162Isup3.cml

Additional supplementary materials:  crystallographic information; 3D view; checkCIF report

## Figures and Tables

**Table 1:** Hydrogen-bond geometry (Å, °) *Cg*1, *Cg*2 and *Cg*3 are the centroids of the C14*A*–C19*A*, N1*B*–N3*B*/C1*B*–C2*B* and C14*B*–C19*B* rings, respectively.

*D*—H⋯*A*	*D*—H	H⋯*A*	*D*⋯*A*	*D*—H⋯*A*
C20*A*—H20*C*⋯N3*A* ^i^	0.96	2.53	3.311 (3)	138
C4*A*—H4*AB*⋯*Cg*2^ii^	0.97	2.98	3.837 (3)	148
C5*B*—H5*BA*⋯*Cg*1^iii^	0.98	2.97	3.833 (11)	147
C13*A*—H13*B*⋯*Cg*1^iii^	0.97	2.68	3.418 (14)	133
C20*B*—H20*F*⋯*Cg*3^iv^	0.96	2.92	3.595 (3)	128
C13*X*—H13*E*⋯*Cg*1^iii^	0.97	2.65	3.42 (2)	137

Symmetry codes: (i) 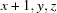; (ii) 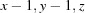; (iii) 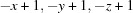; (iv) 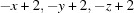.

**Fig. 1.**
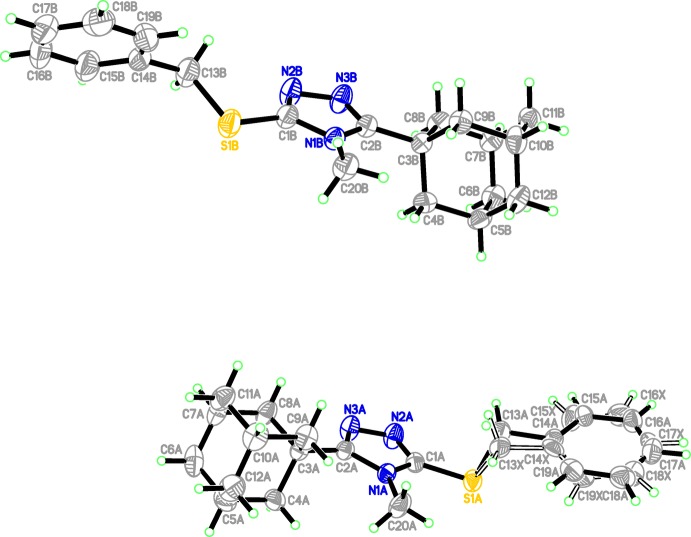
The molecular structure of the title compound, showing 30% probability displacement ellipsoids and the atom-numbering scheme. Open bond show the minor X component.

**Fig. 2.**
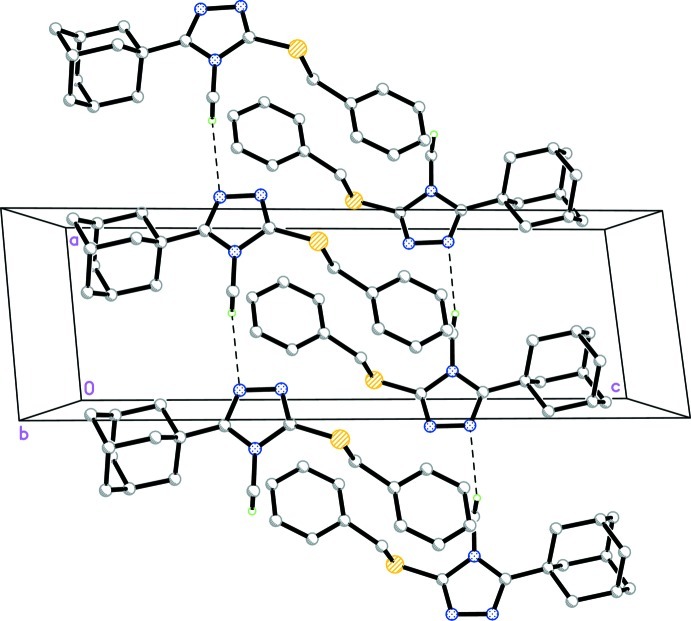
The crystal packing of the title compound viewed along the b axis. Only the major component and H atoms involved with the hydrogen bond were shown. Weak C—H···N interactions are shown as dashed lines.

## supplementary crystallographic information

### Comment

Derivatives of adamantane have long been known for their diverse biological activities including antiviral activity against the influenza (Togo *et al.*, 1968) and HIV viruses (El-Emam *et al.*, 2004). Moreover, adamantane derivatives were recently reported to exhibit marked antibacterial activity (Kadi *et al.*, 2007, 2010). In an earlier publication, we reported the synthesis and potent anti-inflammatory and analgesic activities of a series of 5-(1-adamantyl)-4-substituted-4*H*-1,2,4-triazole-3-thiol derivatives including the title compound (I) (El-Emam & Ibrahim, 1991). We, herein reported the crystal structure of (I).

There are two crystallograpic independent molecules *A* and *B* in the asymmetric unit of the title adamantyl derivative, C_20_H_25_N_3_S (Fig. 1). The whole benzyl group of molecule *A* is disordered over two positions with the refined site-occupancy ratio of 0.63 (2):0.37 (2) for the major and minor components. The 1,2,4-triazole ring is planar with an *r.m.s*. deviation of 0.001 (2) Å for the disordered molecule *A* [0.002 (2) Å for molecule *B*]. The orientation of the benzylsulfanyl moiety with respect to the 1,2,4-triazole ring can be indicated by the dihedral angles between the 1,2,4-triazole and phenyl ring being 24.3 (8) (major component) and 25.8 (13)° (minor component) and the torsion angles C1A–S1A–C13A–C14A = 170.0 (11)° and C1A–S1A–C13X–C14X = -165.8 (13)° for the disordered molecule *A* [the corresponding dihedral and torsion angles are 51.53 (16) and -163.3 (2)° for molecule *B*]. The adamantyl group is planarly attached to the 1,2,4-triazole ring at position 5 or atom C2. The bond distances agree with the literature values (Allen *et al.*, 1987) and are comparable with the related structures (Al-Abdullah *et al.*, 2012; El-Emam *et al.*, 2012; Fun *et al.*, 2011).

In the crystal packing (Fig. 2), the molecules are linked into chains along the *a* axis by weak C—H···N interactions (Table 1). The crystal is further stabilized by weak C—H···π interactions (Table 1).

### Experimental

Sodium methylate (120 mg) was added to a solution of 5-(adamantan-1-yl)-4-methyl-4*H*-1,2,4-triazole-3-thiol (499 mg, 2 mmol) in absolute ethanol (10 ml) and the mixture was heated under reflux for 10 min. Benzyl bromide (342 mg, 2 mmol) was then added and the mixture was heated under reflux for 3 h. On cooling, the mixture was poured onto water (20 ml) and the precipitated crude product was filtered, washed with water and crystallized from ethanol to yield 475 mg (70%) of the title compound as colorless fine needle crystals. Colorless needle-shaped single crystals of the title compound suitable for *X*-ray structure determination were recrystalized from chloroform/ethanol (1:1 *v*/*v*) by the slow evaporation of the solvent at room temperature after several days (m.p. 456–458 K).

### Refinement

All H atoms were placed in calculated positions with C—H = 0.93 Å for aromatic (phenyl), 0.98 Å for aromatic (adamantyl), 0.97 Å for CH_2_ and 0.96 Å for CH_3_ atoms. The *U*_iso_(H) values were constrained to be 1.5*U*_eq_ of the carrier atom for methyl H atoms and 1.2*U*_eq_ for the remaining H atoms. A rotating group model was used for the methyl groups. The whole benzyl group of molecule *A* is disordered over two sites with refined site occupancies of 0.63 (2) and 0.37 (2). Similarity (SAME) restraint was used for both major and minor components of the disordered group.

### Crystal data

**Table:**

C_20_H_25_N_3_S	*Z* = 4
*M**_r_* = 339.49	*F*(000) = 728
Triclinic, *P*1	*D*_x_ = 1.256 Mg m^−^^3^
Hall symbol: -P 1	Melting point = 456–458 K
*a* = 6.4554 (3) Å	Cu *K*α radiation, λ = 1.54178 Å
*b* = 14.0258 (6) Å	Cell parameters from 6505 reflections
*c* = 20.2264 (9) Å	θ = 3.7–69.9°
α = 94.610 (2)°	µ = 1.63 mm^−^^1^
β = 95.568 (3)°	*T* = 296 K
γ = 98.317 (3)°	Needle, colorless
*V* = 1795.23 (14) Å^3^	0.94 × 0.12 × 0.07 mm

### Data collection

**Table:**

Bruker SMART APEXII CCD area-detector diffractometer	6505 independent reflections
Radiation source: fine-focus sealed tube	4522 reflections with *I* > 2σ(*I*)
Graphite monochromator	*R*_int_ = 0.081
φ and ω scans	θ_max_ = 69.9°, θ_min_ = 3.7°
Absorption correction: multi-scan (*SADABS*; Bruker, 2009)	*h* = −6→7
*T*_min_ = 0.310, *T*_max_ = 0.900	*k* = −17→16
22288 measured reflections	*l* = −24→24

### Refinement

**Table:**

Refinement on *F*^2^	Secondary atom site location: difference Fourier map
Least-squares matrix: full	Hydrogen site location: inferred from neighbouring sites
*R*[*F*^2^ > 2σ(*F*^2^)] = 0.052	H-atom parameters constrained
*wR*(*F*^2^) = 0.143	*w* = 1/[σ^2^(*F*_o_^2^) + (0.0644*P*)^2^ + 0.2321*P*] where *P* = (*F*_o_^2^ + 2*F*_c_^2^)/3
*S* = 1.03	(Δ/σ)_max_ = 0.001
6505 reflections	Δρ_max_ = 0.30 e Å^−^^3^
500 parameters	Δρ_min_ = −0.26 e Å^−^^3^
15 restraints	Extinction correction: *SHELXTL* (Sheldrick, 2008), Fc^*^=kFc[1+0.001xFc^2^λ^3^/sin(2θ)]^-1/4^
Primary atom site location: structure-invariant direct methods	Extinction coefficient: 0.0058 (5)

### Special details

**Table:**

Geometry. All esds (except the esd in the dihedral angle between two l.s. planes) are estimated using the full covariance matrix. The cell esds are taken into account individually in the estimation of esds in distances, angles and torsion angles; correlations between esds in cell parameters are only used when they are defined by crystal symmetry. An approximate (isotropic) treatment of cell esds is used for estimating esds involving l.s. planes.
Refinement. Refinement of F^2^ against ALL reflections. The weighted R-factor wR and goodness of fit S are based on F^2^, conventional R-factors R are based on F, with F set to zero for negative F^2^. The threshold expression of F^2^ > 2sigma(F^2^) is used only for calculating R-factors(gt) etc. and is not relevant to the choice of reflections for refinement. R-factors based on F^2^ are statistically about twice as large as those based on F, and R- factors based on ALL data will be even larger.

### Fractional atomic coordinates and isotropic or equivalent isotropic displacement parameters (Å^2^)

**Table:**

	*x*	*y*	*z*	*U*_iso_*/*U*_eq_	Occ. (<1)
S1A	0.13227 (11)	0.28428 (5)	0.53379 (3)	0.0554 (2)	
N1A	0.1834 (3)	0.32730 (13)	0.67079 (9)	0.0416 (4)	
N2A	−0.1139 (3)	0.35694 (18)	0.62025 (10)	0.0612 (6)	
N3A	−0.1062 (3)	0.38366 (18)	0.68787 (10)	0.0584 (6)	
C1A	0.0606 (4)	0.32387 (17)	0.61131 (11)	0.0464 (5)	
C2A	0.0710 (3)	0.36590 (16)	0.71751 (11)	0.0416 (5)	
C3A	0.1320 (3)	0.38416 (16)	0.79177 (11)	0.0409 (5)	
C4A	0.1193 (5)	0.28829 (18)	0.82376 (12)	0.0578 (6)	
H4AA	0.2181	0.2499	0.8058	0.069*	
H4AB	−0.0213	0.2519	0.8133	0.069*	
C5A	0.1715 (6)	0.3083 (2)	0.89991 (13)	0.0725 (8)	
H5AA	0.1644	0.2468	0.9201	0.087*	
C6A	0.0141 (5)	0.3672 (3)	0.92805 (14)	0.0784 (9)	
H6AA	−0.1273	0.3315	0.9180	0.094*	
H6AB	0.0451	0.3790	0.9762	0.094*	
C7A	0.0272 (4)	0.4624 (2)	0.89753 (13)	0.0651 (8)	
H7AA	−0.0744	0.5000	0.9158	0.078*	
C8A	−0.0260 (4)	0.4434 (2)	0.82172 (12)	0.0585 (7)	
H8AA	−0.0205	0.5045	0.8022	0.070*	
H8AB	−0.1677	0.4082	0.8114	0.070*	
C9A	0.3527 (4)	0.4420 (2)	0.81002 (12)	0.0556 (6)	
H9AA	0.3608	0.5034	0.7907	0.067*	
H9AB	0.4557	0.4065	0.7918	0.067*	
C10A	0.4030 (4)	0.4602 (2)	0.88590 (14)	0.0639 (7)	
H10A	0.5455	0.4965	0.8966	0.077*	
C11A	0.2465 (5)	0.5188 (2)	0.91453 (14)	0.0677 (7)	
H11A	0.2790	0.5309	0.9626	0.081*	
H11B	0.2551	0.5806	0.8959	0.081*	
C12A	0.3925 (5)	0.3650 (2)	0.91542 (15)	0.0770 (9)	
H12A	0.4289	0.3761	0.9634	0.092*	
H12B	0.4930	0.3280	0.8970	0.092*	
C13A	0.266 (3)	0.4010 (6)	0.5146 (6)	0.072 (3)	0.63 (2)
H13A	0.1627	0.4437	0.5064	0.087*	0.63 (2)
H13B	0.3635	0.4301	0.5528	0.087*	0.63 (2)
C14A	0.385 (4)	0.391 (3)	0.4541 (7)	0.055 (4)	0.63 (2)
C15A	0.311 (3)	0.4217 (18)	0.3953 (9)	0.053 (2)	0.63 (2)
H15A	0.1837	0.4459	0.3925	0.064*	0.63 (2)
C16A	0.419 (3)	0.4175 (13)	0.3401 (5)	0.057 (3)	0.63 (2)
H16A	0.3656	0.4390	0.3005	0.068*	0.63 (2)
C17A	0.607 (3)	0.3810 (15)	0.3441 (7)	0.068 (4)	0.63 (2)
H17A	0.6836	0.3793	0.3074	0.081*	0.63 (2)
C18A	0.680 (2)	0.3474 (14)	0.4020 (10)	0.067 (3)	0.63 (2)
H18A	0.8012	0.3189	0.4040	0.080*	0.63 (2)
C19A	0.573 (3)	0.3556 (15)	0.4580 (7)	0.063 (4)	0.63 (2)
H19A	0.6289	0.3369	0.4982	0.075*	0.63 (2)
C13X	0.354 (3)	0.3819 (13)	0.5293 (7)	0.058 (3)	0.37 (2)
H13E	0.3137	0.4443	0.5420	0.070*	0.37 (2)
H13F	0.4744	0.3732	0.5594	0.070*	0.37 (2)
C14X	0.407 (7)	0.377 (4)	0.4585 (13)	0.052 (6)	0.37 (2)
C15X	0.293 (5)	0.417 (3)	0.4106 (14)	0.071 (7)	0.37 (2)
H15C	0.1698	0.4399	0.4202	0.086*	0.37 (2)
C16X	0.359 (5)	0.423 (3)	0.3479 (13)	0.073 (7)	0.37 (2)
H16C	0.2839	0.4517	0.3155	0.088*	0.37 (2)
C17X	0.540 (5)	0.386 (2)	0.3339 (11)	0.061 (6)	0.37 (2)
H17C	0.5820	0.3874	0.2913	0.073*	0.37 (2)
C18X	0.657 (4)	0.348 (3)	0.3823 (14)	0.074 (8)	0.37 (2)
H18C	0.7838	0.3284	0.3738	0.088*	0.37 (2)
C19X	0.585 (4)	0.340 (2)	0.4441 (11)	0.053 (5)	0.37 (2)
H19C	0.6560	0.3085	0.4759	0.063*	0.37 (2)
C20A	0.3876 (4)	0.2937 (2)	0.67924 (14)	0.0648 (7)	
H20A	0.4063	0.2549	0.6396	0.097*	
H20B	0.3928	0.2556	0.7166	0.097*	
H20C	0.4978	0.3485	0.6871	0.097*	
S1B	0.81154 (13)	1.08284 (6)	0.97495 (3)	0.0710 (2)	
N1B	0.8218 (3)	1.04770 (14)	0.84119 (9)	0.0455 (4)	
N2B	0.5528 (4)	1.11795 (19)	0.86704 (10)	0.0656 (6)	
N3B	0.5424 (3)	1.10162 (18)	0.79771 (10)	0.0613 (6)	
C1B	0.7201 (4)	1.08489 (19)	0.89102 (12)	0.0526 (6)	
C2B	0.7028 (3)	1.05931 (17)	0.78341 (11)	0.0447 (5)	
C3B	0.7402 (3)	1.02536 (16)	0.71346 (11)	0.0410 (5)	
C4B	0.7354 (4)	0.91500 (17)	0.70604 (13)	0.0534 (6)	
H4BA	0.8480	0.8981	0.7361	0.064*	
H4BB	0.6026	0.8832	0.7181	0.064*	
C5B	0.7617 (4)	0.88001 (18)	0.63401 (12)	0.0558 (6)	
H5BA	0.7602	0.8098	0.6301	0.067*	
C6B	0.5816 (4)	0.90431 (19)	0.58710 (13)	0.0568 (6)	
H6BA	0.5967	0.8816	0.5415	0.068*	
H6BB	0.4483	0.8723	0.5986	0.068*	
C7B	0.5847 (4)	1.01282 (19)	0.59299 (12)	0.0526 (6)	
H7BA	0.4683	1.0284	0.5630	0.063*	
C8B	0.5606 (4)	1.04849 (19)	0.66446 (12)	0.0521 (6)	
H8BA	0.4266	1.0180	0.6765	0.063*	
H8BB	0.5607	1.1179	0.6677	0.063*	
C9B	0.9480 (4)	1.07541 (19)	0.69275 (12)	0.0509 (6)	
H9BA	0.9492	1.1449	0.6959	0.061*	
H9BB	1.0649	1.0623	0.7227	0.061*	
C10B	0.9735 (4)	1.0386 (2)	0.62118 (12)	0.0581 (7)	
H10B	1.1079	1.0704	0.6089	0.070*	
C11B	0.7932 (5)	1.0625 (2)	0.57351 (13)	0.0609 (7)	
H11C	0.7950	1.1320	0.5761	0.073*	
H11D	0.8098	1.0400	0.5280	0.073*	
C12B	0.9713 (4)	0.9301 (2)	0.61595 (13)	0.0620 (7)	
H12C	1.0857	0.9147	0.6460	0.074*	
H12D	0.9912	0.9072	0.5708	0.074*	
C13B	0.6621 (5)	1.1680 (2)	1.01339 (13)	0.0710 (8)	
H13C	0.6613	1.2237	0.9879	0.085*	
H13D	0.5176	1.1376	1.0141	0.085*	
C14B	0.7628 (5)	1.1996 (2)	1.08349 (13)	0.0596 (7)	
C15B	0.6541 (6)	1.1854 (3)	1.13763 (16)	0.0800 (9)	
H15B	0.5155	1.1540	1.1315	0.096*	
C16B	0.7500 (7)	1.2179 (3)	1.20200 (16)	0.0967 (12)	
H16B	0.6737	1.2080	1.2382	0.116*	
C17B	0.9476 (7)	1.2627 (3)	1.21254 (16)	0.0865 (10)	
H17B	1.0083	1.2844	1.2557	0.104*	
C18B	1.0613 (6)	1.2770 (2)	1.15988 (17)	0.0795 (9)	
H18B	1.2001	1.3080	1.1670	0.095*	
C19B	0.9699 (5)	1.2450 (2)	1.09574 (15)	0.0700 (8)	
H19B	1.0491	1.2543	1.0601	0.084*	
C20B	1.0230 (4)	1.0113 (2)	0.85207 (13)	0.0618 (7)	
H20D	1.1267	1.0613	0.8761	0.093*	
H20E	1.0685	0.9926	0.8098	0.093*	
H20F	1.0056	0.9563	0.8775	0.093*	

### Atomic displacement parameters (Å^2^)

**Table:**

	*U*^11^	*U*^22^	*U*^33^	*U*^12^	*U*^13^	*U*^23^
S1A	0.0688 (4)	0.0476 (4)	0.0462 (3)	−0.0050 (3)	0.0160 (3)	−0.0043 (2)
N1A	0.0366 (10)	0.0452 (11)	0.0438 (10)	0.0068 (7)	0.0087 (7)	0.0024 (8)
N2A	0.0521 (13)	0.0891 (17)	0.0431 (12)	0.0203 (11)	−0.0010 (9)	−0.0001 (10)
N3A	0.0450 (12)	0.0905 (17)	0.0419 (11)	0.0249 (10)	−0.0005 (8)	−0.0002 (10)
C1A	0.0489 (14)	0.0468 (14)	0.0423 (12)	0.0023 (10)	0.0068 (10)	0.0034 (10)
C2A	0.0342 (12)	0.0474 (13)	0.0443 (12)	0.0091 (9)	0.0078 (9)	0.0029 (9)
C3A	0.0326 (11)	0.0478 (13)	0.0428 (12)	0.0090 (9)	0.0045 (8)	0.0011 (9)
C4A	0.0772 (18)	0.0483 (15)	0.0455 (14)	0.0044 (12)	0.0030 (12)	0.0038 (11)
C5A	0.112 (3)	0.0550 (17)	0.0461 (15)	0.0053 (16)	−0.0020 (15)	0.0065 (12)
C6A	0.082 (2)	0.100 (3)	0.0439 (15)	−0.0140 (17)	0.0146 (13)	−0.0037 (15)
C7A	0.0567 (16)	0.089 (2)	0.0497 (15)	0.0243 (14)	0.0064 (11)	−0.0146 (14)
C8A	0.0467 (14)	0.0789 (19)	0.0519 (14)	0.0261 (12)	0.0029 (10)	−0.0086 (12)
C9A	0.0371 (13)	0.0664 (17)	0.0594 (15)	−0.0004 (11)	0.0043 (10)	−0.0016 (12)
C10A	0.0424 (15)	0.078 (2)	0.0634 (16)	−0.0011 (12)	−0.0078 (11)	−0.0091 (14)
C11A	0.0755 (19)	0.0649 (18)	0.0576 (16)	0.0122 (14)	−0.0035 (13)	−0.0149 (13)
C12A	0.085 (2)	0.091 (2)	0.0551 (17)	0.0361 (17)	−0.0184 (14)	−0.0039 (15)
C13A	0.113 (8)	0.039 (4)	0.066 (5)	−0.005 (4)	0.044 (5)	0.002 (3)
C14A	0.072 (7)	0.041 (11)	0.049 (8)	−0.003 (4)	0.019 (6)	−0.006 (5)
C15A	0.052 (4)	0.051 (4)	0.060 (6)	0.003 (3)	0.023 (4)	0.012 (5)
C16A	0.062 (9)	0.066 (5)	0.044 (3)	0.011 (6)	0.007 (4)	0.008 (3)
C17A	0.069 (8)	0.079 (6)	0.060 (8)	0.017 (5)	0.024 (6)	0.007 (5)
C18A	0.059 (4)	0.057 (5)	0.086 (9)	0.005 (3)	0.012 (5)	0.014 (6)
C19A	0.090 (7)	0.046 (8)	0.045 (4)	−0.006 (4)	−0.004 (4)	−0.001 (5)
C13X	0.073 (8)	0.039 (7)	0.058 (6)	−0.005 (4)	0.018 (5)	−0.007 (4)
C14X	0.076 (16)	0.028 (11)	0.049 (10)	−0.007 (7)	0.005 (7)	0.011 (6)
C15X	0.065 (9)	0.069 (13)	0.076 (17)	−0.008 (8)	0.028 (10)	−0.009 (12)
C16X	0.066 (14)	0.069 (8)	0.090 (14)	0.021 (9)	0.012 (9)	0.017 (9)
C17X	0.080 (18)	0.059 (8)	0.048 (6)	0.010 (10)	0.015 (10)	0.022 (5)
C18X	0.076 (12)	0.079 (10)	0.072 (15)	0.021 (8)	0.022 (12)	0.009 (10)
C19X	0.069 (9)	0.030 (8)	0.052 (11)	−0.004 (4)	0.000 (8)	−0.005 (8)
C20A	0.0534 (16)	0.085 (2)	0.0619 (16)	0.0331 (14)	0.0120 (12)	−0.0026 (14)
S1B	0.0964 (6)	0.0817 (5)	0.0435 (4)	0.0429 (4)	0.0078 (3)	0.0052 (3)
N1B	0.0460 (11)	0.0507 (12)	0.0414 (10)	0.0135 (8)	0.0042 (8)	0.0032 (8)
N2B	0.0616 (14)	0.0924 (18)	0.0467 (12)	0.0291 (12)	0.0085 (10)	−0.0038 (11)
N3B	0.0516 (13)	0.0874 (17)	0.0479 (12)	0.0280 (11)	0.0031 (9)	−0.0050 (11)
C1B	0.0553 (15)	0.0584 (16)	0.0462 (13)	0.0163 (11)	0.0074 (10)	0.0012 (11)
C2B	0.0399 (12)	0.0504 (14)	0.0441 (12)	0.0103 (9)	0.0024 (9)	0.0024 (10)
C3B	0.0362 (12)	0.0432 (13)	0.0428 (12)	0.0068 (9)	0.0022 (8)	0.0003 (9)
C4B	0.0592 (15)	0.0433 (14)	0.0568 (14)	0.0067 (11)	0.0062 (11)	0.0028 (11)
C5B	0.0675 (16)	0.0426 (14)	0.0556 (15)	0.0101 (11)	0.0053 (12)	−0.0065 (11)
C6B	0.0486 (15)	0.0590 (16)	0.0555 (15)	−0.0034 (11)	−0.0008 (11)	−0.0094 (12)
C7B	0.0501 (14)	0.0594 (16)	0.0458 (13)	0.0122 (11)	−0.0066 (10)	−0.0027 (11)
C8B	0.0475 (14)	0.0574 (15)	0.0506 (14)	0.0158 (11)	−0.0035 (10)	−0.0039 (11)
C9B	0.0431 (13)	0.0530 (15)	0.0509 (14)	−0.0035 (10)	0.0006 (10)	−0.0043 (11)
C10B	0.0463 (14)	0.0742 (19)	0.0476 (14)	−0.0099 (11)	0.0088 (10)	−0.0023 (12)
C11B	0.0711 (18)	0.0626 (17)	0.0440 (14)	−0.0038 (13)	0.0035 (12)	0.0037 (11)
C12B	0.0495 (15)	0.080 (2)	0.0560 (15)	0.0184 (13)	0.0066 (11)	−0.0137 (13)
C13B	0.079 (2)	0.086 (2)	0.0532 (16)	0.0326 (16)	0.0101 (13)	0.0007 (14)
C14B	0.080 (2)	0.0537 (16)	0.0516 (15)	0.0260 (13)	0.0173 (13)	0.0057 (11)
C15B	0.089 (2)	0.084 (2)	0.0680 (19)	0.0085 (17)	0.0321 (17)	−0.0047 (16)
C16B	0.130 (3)	0.105 (3)	0.0550 (19)	0.006 (2)	0.038 (2)	−0.0045 (18)
C17B	0.129 (3)	0.073 (2)	0.0564 (19)	0.021 (2)	0.0066 (19)	−0.0063 (15)
C18B	0.088 (2)	0.064 (2)	0.083 (2)	0.0131 (16)	−0.0019 (17)	0.0020 (16)
C19B	0.085 (2)	0.073 (2)	0.0595 (17)	0.0221 (16)	0.0185 (15)	0.0159 (14)
C20B	0.0616 (17)	0.0743 (19)	0.0538 (15)	0.0314 (13)	−0.0012 (11)	0.0030 (13)

### Geometric parameters (Å, º)

**Table:**

S1A—C1A	1.748 (2)	C17X—H17C	0.9300
S1A—C13A	1.826 (8)	C18X—C19X	1.384 (14)
S1A—C13X	1.845 (15)	C18X—H18C	0.9300
N1A—C1A	1.368 (3)	C19X—H19C	0.9300
N1A—C2A	1.370 (3)	C20A—H20A	0.9600
N1A—C20A	1.462 (3)	C20A—H20B	0.9600
N2A—C1A	1.301 (3)	C20A—H20C	0.9600
N2A—N3A	1.383 (3)	S1B—C1B	1.746 (2)
N3A—C2A	1.305 (3)	S1B—C13B	1.814 (3)
C2A—C3A	1.507 (3)	N1B—C1B	1.364 (3)
C3A—C9A	1.530 (3)	N1B—C2B	1.369 (3)
C3A—C4A	1.534 (3)	N1B—C20B	1.466 (3)
C3A—C8A	1.543 (3)	N2B—C1B	1.303 (3)
C4A—C5A	1.539 (3)	N2B—N3B	1.396 (3)
C4A—H4AA	0.9700	N3B—C2B	1.310 (3)
C4A—H4AB	0.9700	C2B—C3B	1.509 (3)
C5A—C12A	1.520 (5)	C3B—C9B	1.534 (3)
C5A—C6A	1.523 (5)	C3B—C4B	1.539 (3)
C5A—H5AA	0.9800	C3B—C8B	1.539 (3)
C6A—C7A	1.511 (5)	C4B—C5B	1.533 (3)
C6A—H6AA	0.9700	C4B—H4BA	0.9700
C6A—H6AB	0.9700	C4B—H4BB	0.9700
C7A—C11A	1.512 (4)	C5B—C12B	1.523 (4)
C7A—C8A	1.531 (3)	C5B—C6B	1.523 (4)
C7A—H7AA	0.9800	C5B—H5BA	0.9800
C8A—H8AA	0.9700	C6B—C7B	1.514 (4)
C8A—H8AB	0.9700	C6B—H6BA	0.9700
C9A—C10A	1.531 (4)	C6B—H6BB	0.9700
C9A—H9AA	0.9700	C7B—C8B	1.521 (3)
C9A—H9AB	0.9700	C7B—C11B	1.526 (4)
C10A—C12A	1.502 (5)	C7B—H7BA	0.9800
C10A—C11A	1.519 (4)	C8B—H8BA	0.9700
C10A—H10A	0.9800	C8B—H8BB	0.9700
C11A—H11A	0.9700	C9B—C10B	1.529 (3)
C11A—H11B	0.9700	C9B—H9BA	0.9700
C12A—H12A	0.9700	C9B—H9BB	0.9700
C12A—H12B	0.9700	C10B—C12B	1.516 (4)
C13A—C14A	1.516 (8)	C10B—C11B	1.531 (4)
C13A—H13A	0.9700	C10B—H10B	0.9800
C13A—H13B	0.9700	C11B—H11C	0.9700
C14A—C15A	1.362 (10)	C11B—H11D	0.9700
C14A—C19A	1.376 (11)	C12B—H12C	0.9700
C15A—C16A	1.373 (10)	C12B—H12D	0.9700
C15A—H15A	0.9300	C13B—C14B	1.505 (4)
C16A—C17A	1.384 (10)	C13B—H13C	0.9700
C16A—H16A	0.9300	C13B—H13D	0.9700
C17A—C18A	1.365 (11)	C14B—C15B	1.369 (4)
C17A—H17A	0.9300	C14B—C19B	1.385 (4)
C18A—C19A	1.385 (10)	C15B—C16B	1.397 (5)
C18A—H18A	0.9300	C15B—H15B	0.9300
C19A—H19A	0.9300	C16B—C17B	1.327 (5)
C13X—C14X	1.504 (14)	C16B—H16B	0.9300
C13X—H13E	0.9700	C17B—C18B	1.363 (5)
C13X—H13F	0.9700	C17B—H17B	0.9300
C14X—C15X	1.362 (15)	C18B—C19B	1.383 (4)
C14X—C19X	1.374 (14)	C18B—H18B	0.9300
C15X—C16X	1.384 (14)	C19B—H19B	0.9300
C15X—H15C	0.9300	C20B—H20D	0.9600
C16X—C17X	1.385 (14)	C20B—H20E	0.9600
C16X—H16C	0.9300	C20B—H20F	0.9600
C17X—C18X	1.367 (15)		
			
C1A—S1A—C13A	97.2 (2)	C17X—C18X—C19X	119.4 (15)
C1A—S1A—C13X	98.2 (4)	C17X—C18X—H18C	120.3
C1A—N1A—C2A	105.04 (18)	C19X—C18X—H18C	120.3
C1A—N1A—C20A	125.09 (19)	C14X—C19X—C18X	120.1 (15)
C2A—N1A—C20A	129.9 (2)	C14X—C19X—H19C	120.0
C1A—N2A—N3A	106.63 (19)	C18X—C19X—H19C	120.0
C2A—N3A—N2A	108.67 (18)	N1A—C20A—H20A	109.5
N2A—C1A—N1A	110.59 (19)	N1A—C20A—H20B	109.5
N2A—C1A—S1A	124.74 (19)	H20A—C20A—H20B	109.5
N1A—C1A—S1A	124.64 (17)	N1A—C20A—H20C	109.5
N3A—C2A—N1A	109.07 (19)	H20A—C20A—H20C	109.5
N3A—C2A—C3A	123.56 (18)	H20B—C20A—H20C	109.5
N1A—C2A—C3A	127.36 (19)	C1B—S1B—C13B	100.25 (12)
C2A—C3A—C9A	112.91 (18)	C1B—N1B—C2B	104.86 (18)
C2A—C3A—C4A	110.55 (19)	C1B—N1B—C20B	124.2 (2)
C9A—C3A—C4A	109.2 (2)	C2B—N1B—C20B	130.82 (19)
C2A—C3A—C8A	108.14 (18)	C1B—N2B—N3B	106.24 (19)
C9A—C3A—C8A	107.7 (2)	C2B—N3B—N2B	108.07 (19)
C4A—C3A—C8A	108.2 (2)	N2B—C1B—N1B	111.3 (2)
C3A—C4A—C5A	109.9 (2)	N2B—C1B—S1B	127.25 (18)
C3A—C4A—H4AA	109.7	N1B—C1B—S1B	121.46 (18)
C5A—C4A—H4AA	109.7	N3B—C2B—N1B	109.56 (19)
C3A—C4A—H4AB	109.7	N3B—C2B—C3B	124.0 (2)
C5A—C4A—H4AB	109.7	N1B—C2B—C3B	126.43 (19)
H4AA—C4A—H4AB	108.2	C2B—C3B—C9B	113.09 (18)
C12A—C5A—C6A	109.5 (2)	C2B—C3B—C4B	110.45 (19)
C12A—C5A—C4A	109.0 (3)	C9B—C3B—C4B	109.48 (19)
C6A—C5A—C4A	109.6 (3)	C2B—C3B—C8B	108.56 (17)
C12A—C5A—H5AA	109.6	C9B—C3B—C8B	107.48 (19)
C6A—C5A—H5AA	109.6	C4B—C3B—C8B	107.58 (19)
C4A—C5A—H5AA	109.6	C5B—C4B—C3B	110.3 (2)
C7A—C6A—C5A	109.5 (2)	C5B—C4B—H4BA	109.6
C7A—C6A—H6AA	109.8	C3B—C4B—H4BA	109.6
C5A—C6A—H6AA	109.8	C5B—C4B—H4BB	109.6
C7A—C6A—H6AB	109.8	C3B—C4B—H4BB	109.6
C5A—C6A—H6AB	109.8	H4BA—C4B—H4BB	108.1
H6AA—C6A—H6AB	108.2	C12B—C5B—C6B	109.9 (2)
C6A—C7A—C11A	109.5 (3)	C12B—C5B—C4B	109.0 (2)
C6A—C7A—C8A	109.6 (2)	C6B—C5B—C4B	109.5 (2)
C11A—C7A—C8A	110.3 (2)	C12B—C5B—H5BA	109.5
C6A—C7A—H7AA	109.1	C6B—C5B—H5BA	109.5
C11A—C7A—H7AA	109.1	C4B—C5B—H5BA	109.5
C8A—C7A—H7AA	109.1	C7B—C6B—C5B	109.38 (19)
C7A—C8A—C3A	110.15 (19)	C7B—C6B—H6BA	109.8
C7A—C8A—H8AA	109.6	C5B—C6B—H6BA	109.8
C3A—C8A—H8AA	109.6	C7B—C6B—H6BB	109.8
C7A—C8A—H8AB	109.6	C5B—C6B—H6BB	109.8
C3A—C8A—H8AB	109.6	H6BA—C6B—H6BB	108.2
H8AA—C8A—H8AB	108.1	C6B—C7B—C8B	109.8 (2)
C3A—C9A—C10A	110.3 (2)	C6B—C7B—C11B	109.4 (2)
C3A—C9A—H9AA	109.6	C8B—C7B—C11B	109.4 (2)
C10A—C9A—H9AA	109.6	C6B—C7B—H7BA	109.4
C3A—C9A—H9AB	109.6	C8B—C7B—H7BA	109.4
C10A—C9A—H9AB	109.6	C11B—C7B—H7BA	109.4
H9AA—C9A—H9AB	108.1	C7B—C8B—C3B	111.25 (19)
C12A—C10A—C11A	110.1 (3)	C7B—C8B—H8BA	109.4
C12A—C10A—C9A	109.4 (2)	C3B—C8B—H8BA	109.4
C11A—C10A—C9A	109.8 (2)	C7B—C8B—H8BB	109.4
C12A—C10A—H10A	109.2	C3B—C8B—H8BB	109.4
C11A—C10A—H10A	109.2	H8BA—C8B—H8BB	108.0
C9A—C10A—H10A	109.2	C10B—C9B—C3B	110.14 (19)
C7A—C11A—C10A	108.8 (2)	C10B—C9B—H9BA	109.6
C7A—C11A—H11A	109.9	C3B—C9B—H9BA	109.6
C10A—C11A—H11A	109.9	C10B—C9B—H9BB	109.6
C7A—C11A—H11B	109.9	C3B—C9B—H9BB	109.6
C10A—C11A—H11B	109.9	H9BA—C9B—H9BB	108.1
H11A—C11A—H11B	108.3	C12B—C10B—C9B	109.9 (2)
C10A—C12A—C5A	109.8 (2)	C12B—C10B—C11B	109.5 (2)
C10A—C12A—H12A	109.7	C9B—C10B—C11B	109.7 (2)
C5A—C12A—H12A	109.7	C12B—C10B—H10B	109.2
C10A—C12A—H12B	109.7	C9B—C10B—H10B	109.2
C5A—C12A—H12B	109.7	C11B—C10B—H10B	109.2
H12A—C12A—H12B	108.2	C7B—C11B—C10B	108.9 (2)
C14A—C13A—S1A	112.2 (15)	C7B—C11B—H11C	109.9
C14A—C13A—H13A	109.2	C10B—C11B—H11C	109.9
S1A—C13A—H13A	109.2	C7B—C11B—H11D	109.9
C14A—C13A—H13B	109.2	C10B—C11B—H11D	109.9
S1A—C13A—H13B	109.2	H11C—C11B—H11D	108.3
H13A—C13A—H13B	107.9	C10B—C12B—C5B	109.7 (2)
C15A—C14A—C19A	119.0 (8)	C10B—C12B—H12C	109.7
C15A—C14A—C13A	119.9 (11)	C5B—C12B—H12C	109.7
C19A—C14A—C13A	121.0 (11)	C10B—C12B—H12D	109.7
C14A—C15A—C16A	121.5 (8)	C5B—C12B—H12D	109.7
C14A—C15A—H15A	119.2	H12C—C12B—H12D	108.2
C16A—C15A—H15A	119.2	C14B—C13B—S1B	108.66 (19)
C15A—C16A—C17A	119.4 (9)	C14B—C13B—H13C	110.0
C15A—C16A—H16A	120.3	S1B—C13B—H13C	110.0
C17A—C16A—H16A	120.3	C14B—C13B—H13D	110.0
C18A—C17A—C16A	119.5 (10)	S1B—C13B—H13D	110.0
C18A—C17A—H17A	120.2	H13C—C13B—H13D	108.3
C16A—C17A—H17A	120.2	C15B—C14B—C19B	117.2 (3)
C17A—C18A—C19A	120.4 (11)	C15B—C14B—C13B	121.7 (3)
C17A—C18A—H18A	119.8	C19B—C14B—C13B	121.1 (3)
C19A—C18A—H18A	119.8	C14B—C15B—C16B	120.4 (3)
C14A—C19A—C18A	120.0 (10)	C14B—C15B—H15B	119.8
C14A—C19A—H19A	120.0	C16B—C15B—H15B	119.8
C18A—C19A—H19A	120.0	C17B—C16B—C15B	121.4 (3)
C14X—C13X—S1A	108 (3)	C17B—C16B—H16B	119.3
C14X—C13X—H13E	110.2	C15B—C16B—H16B	119.3
S1A—C13X—H13E	110.2	C16B—C17B—C18B	119.8 (3)
C14X—C13X—H13F	110.2	C16B—C17B—H17B	120.1
S1A—C13X—H13F	110.2	C18B—C17B—H17B	120.1
H13E—C13X—H13F	108.5	C17B—C18B—C19B	119.8 (4)
C15X—C14X—C19X	120.4 (14)	C17B—C18B—H18B	120.1
C15X—C14X—C13X	120.8 (18)	C19B—C18B—H18B	120.1
C19X—C14X—C13X	118.6 (17)	C18B—C19B—C14B	121.4 (3)
C14X—C15X—C16X	120.1 (17)	C18B—C19B—H19B	119.3
C14X—C15X—H15C	119.9	C14B—C19B—H19B	119.3
C16X—C15X—H15C	119.9	N1B—C20B—H20D	109.5
C15X—C16X—C17X	119.2 (17)	N1B—C20B—H20E	109.5
C15X—C16X—H16C	120.4	H20D—C20B—H20E	109.5
C17X—C16X—H16C	120.4	N1B—C20B—H20F	109.5
C18X—C17X—C16X	120.6 (15)	H20D—C20B—H20F	109.5
C18X—C17X—H17C	119.7	H20E—C20B—H20F	109.5
C16X—C17X—H17C	119.7		
			
C1A—N2A—N3A—C2A	−0.1 (3)	C13X—C14X—C15X—C16X	172 (4)
N3A—N2A—C1A—N1A	0.3 (3)	C14X—C15X—C16X—C17X	2 (7)
N3A—N2A—C1A—S1A	−177.74 (18)	C15X—C16X—C17X—C18X	−3 (6)
C2A—N1A—C1A—N2A	−0.4 (3)	C16X—C17X—C18X—C19X	5 (6)
C20A—N1A—C1A—N2A	178.5 (2)	C15X—C14X—C19X—C18X	5 (8)
C2A—N1A—C1A—S1A	177.69 (17)	C13X—C14X—C19X—C18X	−170 (4)
C20A—N1A—C1A—S1A	−3.5 (3)	C17X—C18X—C19X—C14X	−6 (6)
C13A—S1A—C1A—N2A	89.2 (6)	C1B—N2B—N3B—C2B	0.3 (3)
C13X—S1A—C1A—N2A	111.7 (7)	N3B—N2B—C1B—N1B	0.2 (3)
C13A—S1A—C1A—N1A	−88.6 (6)	N3B—N2B—C1B—S1B	−178.5 (2)
C13X—S1A—C1A—N1A	−66.1 (7)	C2B—N1B—C1B—N2B	−0.5 (3)
N2A—N3A—C2A—N1A	−0.1 (3)	C20B—N1B—C1B—N2B	175.5 (3)
N2A—N3A—C2A—C3A	−178.8 (2)	C2B—N1B—C1B—S1B	178.19 (18)
C1A—N1A—C2A—N3A	0.3 (3)	C20B—N1B—C1B—S1B	−5.8 (4)
C20A—N1A—C2A—N3A	−178.5 (3)	C13B—S1B—C1B—N2B	−16.1 (3)
C1A—N1A—C2A—C3A	178.9 (2)	C13B—S1B—C1B—N1B	165.4 (2)
C20A—N1A—C2A—C3A	0.1 (4)	N2B—N3B—C2B—N1B	−0.6 (3)
N3A—C2A—C3A—C9A	−129.8 (2)	N2B—N3B—C2B—C3B	176.8 (2)
N1A—C2A—C3A—C9A	51.8 (3)	C1B—N1B—C2B—N3B	0.7 (3)
N3A—C2A—C3A—C4A	107.6 (3)	C20B—N1B—C2B—N3B	−174.9 (3)
N1A—C2A—C3A—C4A	−70.9 (3)	C1B—N1B—C2B—C3B	−176.6 (2)
N3A—C2A—C3A—C8A	−10.7 (3)	C20B—N1B—C2B—C3B	7.7 (4)
N1A—C2A—C3A—C8A	170.8 (2)	N3B—C2B—C3B—C9B	118.8 (3)
C2A—C3A—C4A—C5A	−177.3 (2)	N1B—C2B—C3B—C9B	−64.2 (3)
C9A—C3A—C4A—C5A	58.0 (3)	N3B—C2B—C3B—C4B	−118.1 (3)
C8A—C3A—C4A—C5A	−59.0 (3)	N1B—C2B—C3B—C4B	58.9 (3)
C3A—C4A—C5A—C12A	−59.6 (3)	N3B—C2B—C3B—C8B	−0.3 (3)
C3A—C4A—C5A—C6A	60.2 (3)	N1B—C2B—C3B—C8B	176.7 (2)
C12A—C5A—C6A—C7A	59.1 (3)	C2B—C3B—C4B—C5B	177.00 (19)
C4A—C5A—C6A—C7A	−60.4 (3)	C9B—C3B—C4B—C5B	−57.8 (3)
C5A—C6A—C7A—C11A	−60.6 (3)	C8B—C3B—C4B—C5B	58.7 (3)
C5A—C6A—C7A—C8A	60.5 (3)	C3B—C4B—C5B—C12B	59.6 (3)
C6A—C7A—C8A—C3A	−60.5 (3)	C3B—C4B—C5B—C6B	−60.7 (3)
C11A—C7A—C8A—C3A	60.2 (3)	C12B—C5B—C6B—C7B	−59.6 (3)
C2A—C3A—C8A—C7A	179.0 (2)	C4B—C5B—C6B—C7B	60.2 (3)
C9A—C3A—C8A—C7A	−58.7 (3)	C5B—C6B—C7B—C8B	−59.6 (3)
C4A—C3A—C8A—C7A	59.2 (3)	C5B—C6B—C7B—C11B	60.6 (3)
C2A—C3A—C9A—C10A	178.6 (2)	C6B—C7B—C8B—C3B	59.8 (3)
C4A—C3A—C9A—C10A	−58.0 (3)	C11B—C7B—C8B—C3B	−60.3 (3)
C8A—C3A—C9A—C10A	59.3 (3)	C2B—C3B—C8B—C7B	−178.0 (2)
C3A—C9A—C10A—C12A	59.8 (3)	C9B—C3B—C8B—C7B	59.3 (3)
C3A—C9A—C10A—C11A	−61.1 (3)	C4B—C3B—C8B—C7B	−58.5 (3)
C6A—C7A—C11A—C10A	60.8 (3)	C2B—C3B—C9B—C10B	−179.1 (2)
C8A—C7A—C11A—C10A	−59.9 (3)	C4B—C3B—C9B—C10B	57.3 (3)
C12A—C10A—C11A—C7A	−60.4 (3)	C8B—C3B—C9B—C10B	−59.3 (3)
C9A—C10A—C11A—C7A	60.1 (3)	C3B—C9B—C10B—C12B	−59.2 (3)
C11A—C10A—C12A—C5A	59.5 (3)	C3B—C9B—C10B—C11B	61.3 (3)
C9A—C10A—C12A—C5A	−61.3 (3)	C6B—C7B—C11B—C10B	−60.9 (3)
C6A—C5A—C12A—C10A	−58.6 (3)	C8B—C7B—C11B—C10B	59.5 (3)
C4A—C5A—C12A—C10A	61.3 (3)	C12B—C10B—C11B—C7B	60.4 (3)
C1A—S1A—C13A—C14A	170.0 (11)	C9B—C10B—C11B—C7B	−60.3 (3)
C13X—S1A—C13A—C14A	75.9 (17)	C9B—C10B—C12B—C5B	60.9 (3)
S1A—C13A—C14A—C15A	107 (3)	C11B—C10B—C12B—C5B	−59.6 (3)
S1A—C13A—C14A—C19A	−75 (3)	C6B—C5B—C12B—C10B	59.2 (3)
C19A—C14A—C15A—C16A	0 (5)	C4B—C5B—C12B—C10B	−60.9 (3)
C13A—C14A—C15A—C16A	178 (2)	C1B—S1B—C13B—C14B	−163.3 (2)
C14A—C15A—C16A—C17A	0 (4)	S1B—C13B—C14B—C15B	−121.0 (3)
C15A—C16A—C17A—C18A	2 (3)	S1B—C13B—C14B—C19B	59.6 (3)
C16A—C17A—C18A—C19A	−4 (3)	C19B—C14B—C15B—C16B	1.3 (5)
C15A—C14A—C19A—C18A	−2 (5)	C13B—C14B—C15B—C16B	−178.2 (3)
C13A—C14A—C19A—C18A	180 (2)	C14B—C15B—C16B—C17B	−0.2 (6)
C17A—C18A—C19A—C14A	5 (4)	C15B—C16B—C17B—C18B	−0.6 (6)
C1A—S1A—C13X—C14X	−165.8 (13)	C16B—C17B—C18B—C19B	0.3 (5)
C13A—S1A—C13X—C14X	−76.9 (18)	C17B—C18B—C19B—C14B	0.8 (5)
S1A—C13X—C14X—C15X	81 (6)	C15B—C14B—C19B—C18B	−1.5 (4)
S1A—C13X—C14X—C19X	−104 (5)	C13B—C14B—C19B—C18B	177.9 (3)
C19X—C14X—C15X—C16X	−3 (9)		

### Hydrogen-bond geometry (Å, º)

*Cg*1, *Cg*2 and *Cg*3 are the centroids of the C14A–C19A, N1B–N3B/C1B–C2B and C14B–C19B rings, respectively.

**Table:**

*D*—H···*A*	*D*—H	H···*A*	*D*···*A*	*D*—H···*A*
C20*A*—H20*C*···N3*A*^i^	0.96	2.53	3.311 (3)	138
C4*A*—H4*AB*···*Cg*2^ii^	0.97	2.98	3.837 (3)	148
C5*B*—H5*BA*···*Cg*1^iii^	0.98	2.97	3.833 (11)	147
C13*A*—H13*B*···*Cg*1^iii^	0.97	2.68	3.418 (14)	133
C20*B*—H20*F*···*Cg*3^iv^	0.96	2.92	3.595 (3)	128
C13*X*—H13*E*···*Cg*1^iii^	0.97	2.65	3.42 (2)	137

Symmetry codes: (i) *x*+1, *y*, *z*; (ii) *x*−1, *y*−1, *z*; (iii) −*x*+1, −*y*+1, −*z*+1; (iv) −*x*+2, −*y*+2, −*z*+2.
